# Editorial: The therapeutic inhibition of macrophage checkpoints

**DOI:** 10.3389/fimmu.2024.1418281

**Published:** 2024-04-26

**Authors:** Alexandre P. A. Theocharides, Gregor Hutter, Yasuyuki Saito

**Affiliations:** ^1^ Department of Medical Oncology and Hematology, University of Zurich and University Hospital Zurich, Zurich, Switzerland; ^2^ Departments of Neurosurgery and Biomedicine, University of Basel and University Hospital Basel, Basel, Switzerland; ^3^ Division of Molecular and Cellular Signaling, Department of Biochemistry and Molecular Biology, Kobe University Graduate School of Medicine, Kobe, Japan

**Keywords:** macrophages, immune checkpoints, cd47, immunotherapy, signal regulatory protein alpha

## Introduction

Cancer cells escape the immune system through interactions with immune checkpoints. While inhibiting interactions between T and tumor cells has become a cornerstone in the therapy of solid tumors, less is known about the therapeutic relevance and potential of blocking tumor cell interactions with other cellular components of the immune system. In particular, inhibition of macrophage checkpoints in solid and hematological malignancies is a rapidly developing field. Specifically, blocking the interaction between the immunoglobulin superfamily member CD47 on cancer cells and the inhibitory macrophage receptor SIRPα is the most advanced therapeutic approach currently investigated in multiple clinical trials. In addition, other macrophage checkpoints such as the CD24-Siglec10 and the MHC-LILRB1 interactions exist and may also be clinically exploited. In this Research Topic, we cover a collection of research articles about the most recent, cutting-edge developments in leveraging macrophage checkpoints to fight cancer.

## Structure of macrophage immune checkpoints

Ubil and Zahid review the data of Mer (MerTK) as an innate immune checkpoint in macrophages. Mer has a similar structure to Tyro3 and Axl, and several functional domains, which makes Mer signaling a complex process. The authors describe structural features of Mer, and its role as an innate immune checkpoint. Importantly, they provide an overview of therapeutic strategies targeting Mer in cancer. Mer-directed therapeutics are in various stages of development, with some already in clinical trials. There are antibody-based approaches to targeting Mer and compounds that directly inhibit Mer kinase. Both strategies have been investigated in preclinical models, which are described in this review. Furthermore, MER is a biomarker for therapy resistance likely due to its various roles in the suppression of anti-tumor response. Finally, the review summarizes ongoing clinical trials with Mer inhibition in various cancers.

Zhang et al. describe the structural-functional diversity of CD47 isoforms. CD47 is a ubiquitously expressed transmembrane glycoprotein. It participates in various important physiological cell functions, including phagocytosis, apoptosis, proliferation, adhesion, and migration, through interactions with its ligands, including the inhibitory receptor signal regulatory protein α (SIRPα), secreted glycoprotein thrombospondin-1 (TSP-1), and integrins. Elevated expression of CD47 is observed in a wide range of cancer cells as a mechanism for evading the immune system and confers poor survival. Therapeutically blocking the interaction between the CD47 and SIRPα is the most advanced therapeutic approach that leverages macrophage immune checkpoints. Multiple clinical trials are currently ongoing, but some trials in acute myeloid leukemia and myelodysplastic syndrome were stopped due to futility. However, there is a large cohort of CD47 proteins with cell-, tissue-, and temporal-specific expression and functional profiles. These profiles have been derived from a single gene through alternative splicing and post-translational modifications. In this review the origins and molecular properties of CD47 proteoforms and their roles under physiological and pathological conditions are explored. These findings contribute to the understanding of CD47 proteoform diversity and identification of novel clinical targets and immune-related therapeutic candidates.

## Targeting signal regulatory protein family in cancer

An alternative approach to inhibiting the CD47-SIRPα interaction is targeting its receptor SIRPα on macrophages. Anti-SIRPα antibodies are thought to have a strong potential as a macrophage–targeted cancer immunotherapy, which induces inhibition of the signal but also promotes reprogramming of tumor-associated macrophages (TAMs) in the tumor microenvironment. However, the way to evaluate the effect of human macrophage–targeted cancer immunotherapy has not been established due to the lack of appropriate models using xenograft tumor models. Saito et al. aimed to establish a preclinical cancer immunotherapy model targeting human TAMs *in vivo* using humanized immune system (HIS) mice. The model is generated by the injection of human CD34+ hematopoietic stem and progenitor cells into severe immunodeficient mice expressing human multiple cytokines (HIS-MITRG) mice. Using HIS-MITRG mice, the authors demonstrate the anti-tumor effect of anti-human SIRPα antibodies in combination with rituximab against B-cell lymphoma *in vivo* in a macrophage-dependent manner. The authors also revealed activation of TAMs by RNA-seq analysis after the treatment with an anti-SIRPα antibody in addition to the promotion of phagocytosis by the inhibition of the CD47-SIRPα axis.

Signal regulatory protein (SIRP) is a family member protein including inhibitory (SIRPα) and activating (SIRPβ) receptors. In humans, SIRPβ2 is an orphan Ig-like receptor preferentially expressed on human myeloid lineage cells and is thought to positively regulate their functions in association with the immunoreceptor tyrosine-based activation motif (ITAM)-containing adapter molecule DAP12. However, the precise function of SIRPβ2 in tumor immunity remained unknown. In this Research Topic, Visser et al. revealed a yet uninvestigated SIRP family member, SIRP-beta 2 (SIRP-β2), which is strongly expressed under normal physiological conditions in macrophages and granulocytes. Importantly, SIRP-β2 expression on granulocytes correlated with trogocytosis of cancer cells. Therefore, SIRP-β2 is a novel positive regulator of innate anticancer immunity and a potential costimulatory target for innate immunotherapy.

## Novel macrophage immune checkpoints in cancer immunotherapy

Beyond the CD47-SIRPα axis, many other macrophage checkpoints have been identified recently. In the frame of this Research Topic, the work by Daunke et al. studies the role of the PD1/PD-L1 axis as a potential myeloid checkpoint in pancreatic adenocarcinoma and their liver metastasis. Although the authors did not observe an effect of the immune checkpoint inhibitor durvalumab on macrophages in their 3D spheroid models, they provide a collection of patient-matched primary tumor and their metastasis and can correlate high macrophage-specific PD-L1 expression with metastatic progression. This work partly corroborates findings from Gordon et al. (1) but also denotes the importance of spatial interaction among various players of the immune microenvironment. The team of Lustig et al. elaborated on the role of the Siglec-9-Sialic acid axis in tumor-associated neutrophils. This important innate immune checkpoint has been recently identified in tumor-associated macrophages of solid tumors, and its blockade led to improved survival in preclinical models. Neutrophils are an abundant myeloid cell population within the tumor microenvironment. Lustig et al. showed that desialylation or blockade of Siglec-9 enhanced the efficacy of therapeutic antibodies such as anti-HER2 and anti-EGFR in breast cancer models. They convincingly attribute this effect to tumor-associated neutrophils and could show that antibody-mediated cellular cytotoxicity (ADCC) in neutrophils is improved after Siglec-9-Sialoglykan disruption. This work opens another therapeutic possibility in the armamentarium against difficult-to-treat solid cancers. Lastly, Zeller et al. highlight leukocyte immunoglobulin-like receptor subfamily B member 1 (LILRB1) as a novel macrophage immune checkpoint receptor and concisely review the current state of research and perspectives anti-LILRB strategies.

## Conclusions

This Research Topic shows that the study of established innate immune checkpoints such as CD47 constantly evolves, and novel aspects such as CD47 splice variants need consideration in the future. Beyond CD47, novel macrophage, and in general myeloid checkpoints have arisen that will have a major therapeutic relevance in cancer immunotherapy.

## Author contributions

AT: Writing – original draft, Writing – review & editing. GH: Writing – original draft, Writing – review & editing. YS: Writing – original draft, Writing – review & editing.
